# Growing Trans‐Species Islets in Tumor Extract‐Remodeled Testicles

**DOI:** 10.1002/advs.201801694

**Published:** 2019-01-13

**Authors:** Zhenzhen Wang, Xiaying Rui, Junni Qiu, Yiqing Yan, Jingjing Gan, Shang Liu, Lintao Wang, Junfeng Zhang, Chunming Wang, Lei Dong

**Affiliations:** ^1^ Nanjing Drum Tower Hospital the Affiliated Hospital of Nanjing University Medical School Nanjing Jiangsu 210093 China; ^2^ State Key Laboratory of Pharmaceutical Biotechnology School of Life Sciences Nanjing University Nanjing Jiangsu 210023 China; ^3^ State Key Laboratory of Quality Research in Chinese Medicine Institute of Chinese Medical Sciences University of Macau Taipa 999078 Macau SAR

**Keywords:** diabetes, immune tolerance, testicles, tumor extract, xenogeneic cancer cells

## Abstract

Although pancreatic islet transplantation holds promise for the treatment of type I diabetes, its application has been significantly hampered by transplant rejection. Here, an approach is demonstrated to support trans‐species islet beta cells from a rat to grow and function in the body of a mouse host while overcoming graft rejection. This approach, which builds on remodeling of the mouse testicle by local injection of a tumor homogenate, establishes an immunosuppressive and proregenerative niche in the testicle. This remodeling proves necessary and effective in shaping the testicle into a unique site to accommodate xenograft cells. Rat pancreatic beta cells—from both the insulinoma (cancer cells) and pancreatic islet (normal tissue)—survive, grow, and form a desirable morphology in the remodeled mouse testicle. Notably, when hyperglycemia is induced in the host body, these xenografts secrete insulin to regulate the blood glucose level in mice for as long as 72 days. Furthermore, no graft rejection, acute inflammation, or safety risks are observed throughout the study. In summary, it is demonstrated that the growth of xenogeneic insulinoma cells in a mouse testicle might serve as an alternative approach for islet transplantation.

## 1 Introduction

Type 1 diabetes affects tens of millions of people worldwide and has no cure to date. Pancreatic islet transplantation may be a promising mode of treatment. Studies have shown that transplantation of stem cell‐derived beta‐like cells or rat islets into diabetic mice could control blood glucose levels for up to 21 days.^1^, ^2^ However, these approaches face many obstacles, and the most significant one is transplant rejection.^3^ The host immune system rejects “nonself” cells or tissue, resulting in early graft loss or a lifelong reliance on immunosuppressive drugs with severe side effects.^4^, ^5^ Attempts have been made to isolate transplanted islet cells with biomaterials such as macro/microcapsules,^6^, ^7^ which could effectively prevent the allo/xenogeneic cells from contacting the host tissue and could prove useful in protecting the beta cells from immune attack.^8^, ^9^ However, such encapsulation also cuts off nutrient supply from the body, causing the death of the transplanted cells, or triggers foreign body reactions (FBR), another type of host immune response, leading to fibrosis around the implants.^10^, ^11^ Therefore, for islet transplantation, it remains an unsolved challenge to minimize immune rejection while enabling host connection.

To address this challenge, we considered the use of an existing organ—the testicle—to accommodate islet transplantation. We chose the testicle based on its unique physiological characteristics. In the testicle, a unique combination of immune cells and immunomodulatory factors forms an “immunologically privileged” niche^12^ that protects the survival of allogeneic or xenogeneic cells.^13^, ^14^, ^15^ Compared with artificial scaffolds, the testicle does not have issues such as immune rejection, FBR, and lack of nutrient supply. Additionally, its size is proper for growing enough pancreatic cells to exert glucose‐control function. Additionally, when one testicle is used for accommodating cell transplantation, the other testicle remains functional. Thus, utilizing a testicle as a site for islet engraftment is feasible and desirable.

However, evidence has shown that the immune tolerance of the testicle is not permanent and does not last long enough to support the full function of the transplanted islets.^16^, ^17^, ^18^ First, acute rejection could still occur and fail the transplants in the testicle;^13^, ^17^, ^19^ even if the transplanted islets settled in the testicle, most (13/16, or 81%) of them completely failed to regulate blood glucose.^20^, ^21^ Hence, it is vital to devise strategies that can maintain the immunosuppressive feature of the testicle, which will maximize the therapeutic functions of the grafted islet cells.

**Scheme 1:**
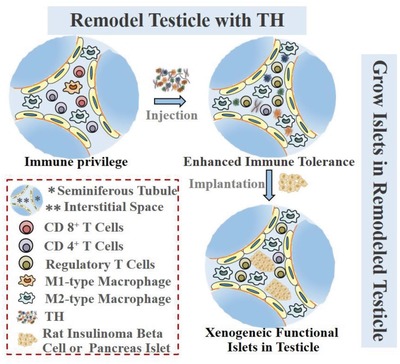
Schematic diagram of the process of remodeling the testicle into a functional islet by utilizing the tumor extract (TH). A mouse testicle can be remodeled into a unique site for growing trans‐species islet beta cells after local injection of tumor‐derived extract therein. The remodeled site showed enhanced immune tolerance with significant increases in antiinflammatory macrophages and Treg cells and decrease in cytotoxic CD 8^+^ T cells. Such a niche enables both cancerous insulinoma cells and pancreas islet cells to effectively control blood glucose.

In this study, as shown in the **Scheme** **1**, we propose to remodel the testicle in mice for the growth of trans‐species (rat) islet cells. To achieve this goal, we first remodeled one testicle by injecting a soluble extract from the tumor homogenate (TH), which we previously showed could protect the implanted xenogeneic cancer cells (XCCs) from rejection in immunocompetent mice.^22^ Then, we transplanted either rat insulinoma beta cells (INS‐1 cells, known for their higher proliferation rate)^23^ or primary rat islet cells into the TH‐remodeled mouse testicle and examined their long‐term survival, response to glucose, and insulin‐producing functions in the host body.^22^, ^24^ The results validated our hypothesis that the TH‐remodeled testicle provided an enhanced immunosuppressive microenvironment to support the growth of xenografted islets, which exerted prolonged antidiabetic function and minimized graft rejection.

## 2 Results

### 2.1 TH Remodeling Enhances the Immunosuppressive Environment in the Mouse Testicle

We employed a TH to enhance the immunosuppressive environment in the testicle. We prepared the TH from a soluble extract of murine sarcoma (grown from an S180 cell graft) and profiled its protein composition by liquid chromatography‐mass spectrometry (LC‐MS) (Table S1, Supporting Information). Both pathway analysis (Reactome Database, Figure S1A and Table S2, Supporting Information) and hierarchical cluster analysis (Gene Ontology Database, Figure S1B, Supporting Information) indicated that the TH components were closely associated with immune activities.

**Figure 1:**
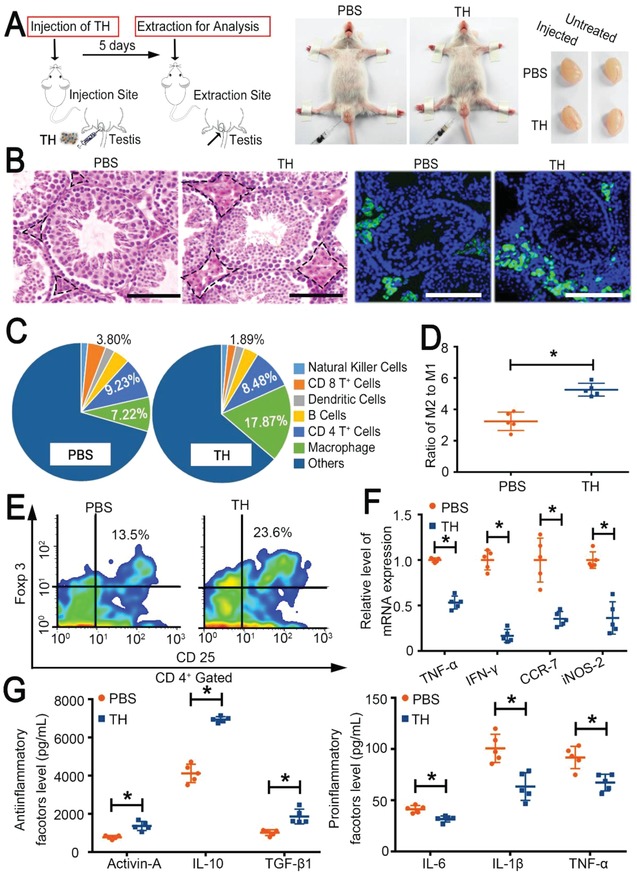
TH remodeling is necessary for establishing an immunosuppressive environment in the mouse testicle. A) Schematic diagram of one testicle injected with TH, with the gross view of the injection shown in the middle and that of the collected testicles shown on the right. B) Representative histological H&E staining of testicles 5 days after a single injection with TH (500 µL kg^−1^) or PBS. Black dotted lines indicate the cells located in the interstitial space. Representative fluorescence photographs of macrophages in the interstitial space are shown on the right. F4/80, a typical marker of macrophages, is shown in green. The blue DAPI‐stained nucleus indicates the cell number and position in the photographs; scale bar = 100 µm. C) Pie charts of the populations of the main immune cells, including natural killer cells, CD 8^+^ T cells, dendritic cells, B cells, CD 4^+^ T cells, and macrophages, according to representative FACS analysis. D) Ratio of M2/M1 macrophage cells calculated by dividing the number of CD 206^+^/F4/80^+^ cells by that of CD 86^+^/F4/80^+^ cells in the testicles treated as shown in panel (A). E) Representative FACS analysis of CD 25^+^/Foxp3^+^ Tregs (CD 4^+^ T cells gated) in the testicles treated as shown in panel (A). F) qRT‐PCR analysis of the expression of key genes related to T cells (TNF‐α and IFN‐γ) or macrophages (CCR‐7 and iNOS‐2) in the testicles treated as shown in panel (A). G) The levels of antiinflammatory (activin A, IL‐10, and TGF‐β1) and proinflammatory (IL‐6, IL‐1β, and TNF‐α) cytokines measured by ELISA in the testicles treated as shown in panel (A). The results are shown as the mean ± SD (*n* = 5 mice per group). *p < 0.05 after ANOVA with Dunnett's tests.

To determine how the TH could change the immune context in the testicle, we injected the TH into one testicle and analyzed the tissue after 5 days (**Figure** **1**A). The TH treatment induced the infiltration of more cells, notably macrophages, into the interstitial space without affecting the appearance of the internal tissue structure of the testicle; the secretion of testosterone also remained normal (Figure 1A,B; Figure S2, Supporting Information). These data demonstrated the safety of the TH injection. Further analysis of the immune cell population revealed that the TH treatment changed the cell proportions (Figure 1C; Figure S3, Supporting Information). In agreement with the immunofluorescence (IF) staining result, the ratio of macrophages significantly increased to 17.87%, ≈2.5 times higher than that of the phosphate buffered saline (PBS) group. For macrophages, both the proportion and number of antiinflammatory macrophages (M2‐type) increased in the TH‐treated group (Figure 1D; Figure S4, Supporting Information), reflecting a more suppressive immune microenvironment.^25^ In addition, even though the number of CD 4^+^ T cells in the TH group remained similar to that in the PBS group, the proportion of regulatory T cells (Tregs; CD 25^+^/Foxp3^+^) in the TH group, which are key mediators of immune suppression,^26^ reached approximately two times that in the PBS‐treated group (23.6% vs 13.5%) (Figure 1E). More importantly, the proportion of cytotoxic CD 8^+^ T cells, a main threat to the long‐term survival of transplanted cells in the testicle,^27^ was dramatically decreased to 1.89%, nearly half of that in the PBS group.

Furthermore, the transcriptional levels of both tumor necrosis factor‐α (TNF‐α)/interferon‐γ (IFN‐γ) and CC chemokine receptor‐7 (CCR‐7)/inducible nitric oxide synthase (iNOS‐2), which are important genes in the immune activation of T cells and macrophages, respectively,^28^, ^29^ decreased after TH injection (Figure 1F). Studies have reported that the production of immunosuppressive and antiinflammatory cytokines, such as transforming growth factor‐beta 1 (TGF‐β1), activin A, and interleukin‐10 (IL‐10), is also important for the immunological privilege.^12^, ^30^, ^31^ Our data showed that these cytokines were indeed upregulated in the testicle upon TH treatment, accompanied by a decrease in the expression of proinflammatory cytokines (IL‐6, IL‐1β, and TNF‐α) (Figure 1G).

These results suggested that the TH treatment strengthened the immunosuppressive characteristics of the testicle tissue. We speculated that this remodeling process would be vital to maintaining long‐term immunotolerance in the testicle, which is necessary for the allo/xenogeneic cells to grow.

### 2.2 TH Remodeling Creates an Immunotolerant Environment in Mice Testicles for Xenotransplantation

**Figure 2:**
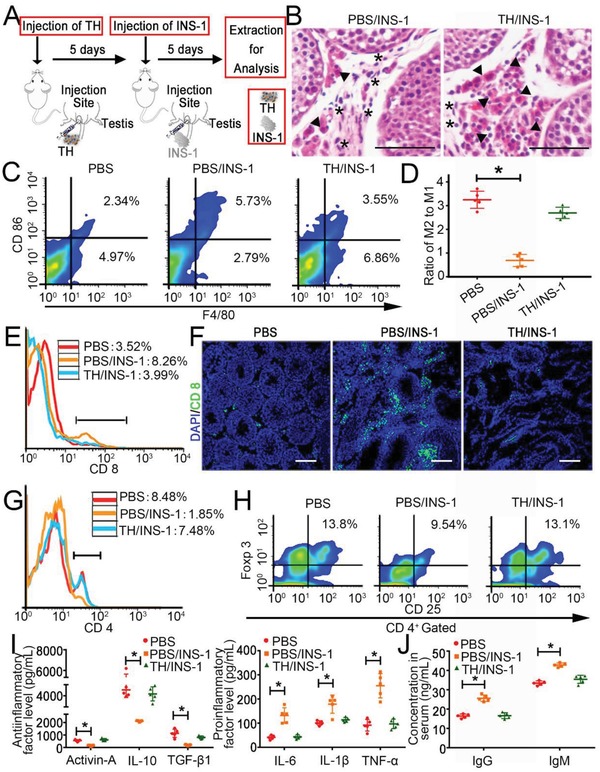
TH remodeling creates an immunotolerant environment in mouse testicles for INS‐1 cell xenotransplantation. A) Schematic diagram of the injection of rat INS‐1 cells into one testicle. B) Representative histological H&E staining of testicles on day 5 after a single injection of INS‐1 cells. Asterisks indicate infiltrated inflammatory cells, and arrows show xenogeneic INS‐1 cells. Scale bar = 50 µm. C) Representative FACS analysis of CD 86^+^/F4/80^+^ cells in the testicles treated as shown in panel (A). D) Proportion of M2/M1 macrophages calculated by dividing the number of CD 206^+^/F4/80^+^ cells by that of CD 86^+^/F4/80^+^ cells. E) Representative FACS analysis of CD 8^+^ T cells in the testicles treated as shown in panel (A). F) Representative fluorescence photographs of CD 8^+^ T cells in the testicles treated as shown in panel (A). Scale bar = 100 µm. G) The percentage of CD 4^+^ T cells in testicles in different groups. H) Representative FACS analysis of CD 25^+^/Foxp3^+^ Treg (CD 4^+^ T cells gated) in different groups. I) The levels of antiinflammatory (activin A, IL‐10, and TGF‐β1) and proinflammatory (IL‐6, IL‐1β, and TNF‐α) cytokines measured by ELISA in the testicles treated as shown in panel (A). J) Quantification of IgG and IgM in the serum of mice 5 days after the injection of INS‐1 cells. The results are shown as the mean ± SD (*n* = 5 mice per group). *p < 0.05 after ANOVA with Dunnett's tests.

To validate whether the TH‐remodeled testicle was capable of accommodating trans‐species cell grafts, we transplanted rat INS‐1 cells to the mouse testicle pretreated with TH or PBS. Five days later, we examined the occurrence of immune rejection or tolerance to these rat cells in the mice (**Figure** **2**A).

First, histological analysis (H&E staining) demonstrated that INS‐1 cells triggered a significant immune response in the PBS‐treated testicle, with abundant inflammatory cells infiltrating into the testicle; however, the rat cells settled down in the TH‐remodeled testicle, with no obvious sign of rejection or acute inflammation (Figure 2B). We assessed the phenotype of macrophages, which play a key role in organizing the innate immune response, and found that the number of proinflammatory (M1) macrophages (CD 86^+^/F4/80^+^) significantly increased upon the inoculation of INS‐1 cells into the PBS‐treated testicles; meanwhile, antiinflammatory (M2) macrophages were present in the TH‐remodeled testicle, similar to the situation in the untreated testicle (Figure 2C,D; Figure S5, Supporting Information). These findings suggest that TH remodeling suppresses the innate immune response against xenograft cells.

Second, we investigated the changes in T cell populations and cytokine expression. Both Fluorescence Activated Cell Sorter (FACS) analysis and IF staining highlighted the cytotoxic immune response in the PBS group, in which the ratio of CD 8^+^ T cells increased to 8.26%, in contrast to 3.99% in the TH‐treated sample and 3.52% in the normal testicle (Figure 2E,F). In addition, the number of CD 4^+^ T cells decreased sharply in the PBS group but not in the TH group (1.85% vs 7.48%, Figure 2G). Notably, the proportion of Treg cells, a key mediator of immune suppression, further decreased in the PBS‐treated testicle upon INS‐1 cell grafting (9.54% in 1.85% CD 4^+^ T cells vs 13.8% in 8.48% CD 4^+^ T cells of the normal group), reflecting the triggering of acute inflammation; however, this decrease did not occur in the TH‐remodeled mice (13.1% in 7.48% CD 4^+^ T cells, Figure 2H). Further cytokine profiling showed that INS‐1 cell implantation significantly increased the level of proinflammatory factors (IL‐6, IL‐1β, and TNF‐α) and decreased that of antiinflammatory factors (activin A, TGF‐β1, and IL‐10) in the PBS‐treated testicle, whereas TH remodeling successfully protected the testicle from such turbulence (Figure 2I). These data indicate that TH treatment effectively established immune tolerance in the testicle, which is an important basis for overcoming transplant rejection.

Third, we observed the systematic immune rejection of INS‐1 cells. The quantification of cytokines in peripheral blood revealed an intense early rejection reduced by the rat cells transplanted into the PBS‐treated mice, as evidenced by a sharp change in the production of IgG and IgM.^32^ However, these changes were much lower in the TH‐treated group (Figure 2J). Furthermore, specific IF staining for INS‐1 cells showed that the transplanted rat cells were confined within the testicle, with no leakage or spreading out of these cells to any other tissues in the host mice (Figure S6, Supporting Information). These findings are crucial for the safe use of TH.

The above results demonstrate that the TH treatment is capable of remodeling the testicle tissue into an immunotolerant environment, and this remodeling is necessary for the survival and function of trans‐species cell transplants. Without using TH, strong immune rejection could still be triggered by the xenograft cells—despite some earlier observations that the testicle exhibited “immunological privilege” to some extent.^12^, ^14^, ^15^

### 2.3 Transplanted Rat Beta Cells Grow in TH‐Remodeled Mouse Testicle

**Figure 3:**
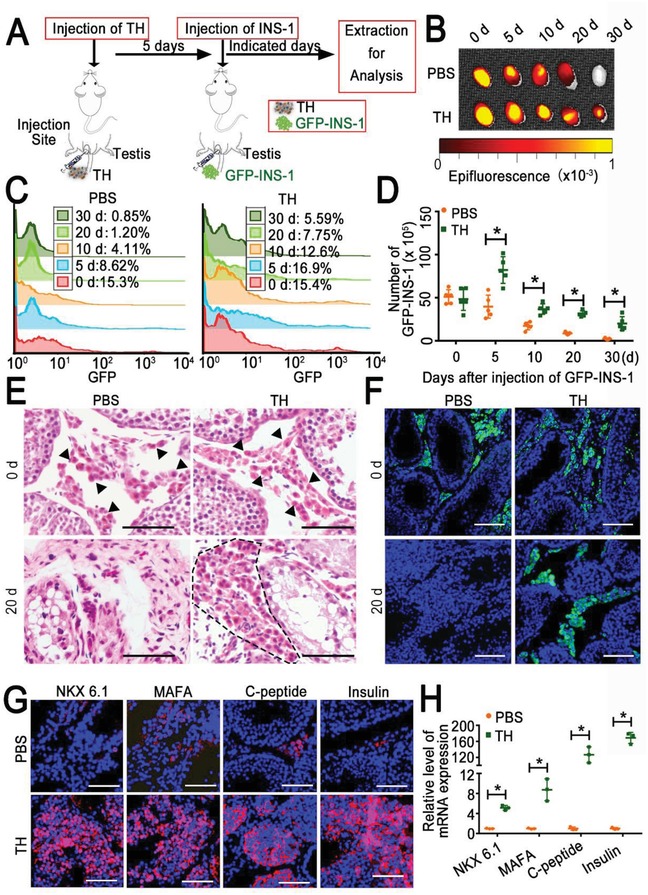
Transplanted rat INS‐1 cells grow in TH‐remodeled mouse testicles. A) Schematic diagram of INS‐1 cell injection into one testicle for growth analysis. B) Representative bioluminescence images of TH‐remodeled or PBS‐treated testicles injected with GFP‐INS‐1 cells on the indicated days. C) FACS analysis of GFP‐positive cells in two groups treated as shown in panel (B). D) Quantitative analysis of GFP‐positive INS‐1 cells calculated by multiplying the total cell number and the percentage of GFP‐INS‐1‐positive cells (*n* = 5 per group). E) Histological analysis (H&E) of testicles 0 or 20 days after intratesticular inoculation of GFP‐INS‐1 cells into TH‐remodeled or PBS‐treated testicles. The black arrows show the initial injected INS‐1 cells. The black dotted line indicates the islet‐like cell clusters. Scale bar = 200 µm. F) Representative fluorescence photographs of GFP‐INS‐1 cells in the testicles of two groups treated as shown in panel (E). G) Representative fluorescence photographs for the beta‐cell transcription factors (NKX 6.1, MAFA, C‐peptide, and insulin) in the testicles treated as shown in panel (E). NKX 6.1, MAFA, C‐peptide and insulin, typical markers of beta cells, are shown in red. Scale bar = 50 µm. H) qRT‐PCR analysis of the transcriptional level of key genes related to INS‐1 cells (NKX 6.1, MAFA, C‐peptide, and insulin) in the testicles treated as shown in panel (E). The results are shown as the mean ± SD (*n* = 3 mice per group). *p < 0.05 after ANOVA with Dunnett's tests.

We next assessed the long‐term survival and growth of these xenograft cells in the host. First, by using a lentivirus, we stably transfected the rat INS‐1 cells with a green fluorescent protein (GFP or GFP‐INS‐1) and injected the GFP‐INS‐1 cells (5 × 10^6^) into the PBS‐treated or TH‐remodeled testicle in mice (**Figure** **3**A). Bioluminescence imaging lasting for 30 days demonstrated that the transplanted cells in the TH‐remodeled testicle could survive throughout the entire period, but those in the PBS group died rapidly, with 59.08% of the signal lost after 20 days (Figure 3B; Figure S7, Supporting Information). This finding was further confirmed by flow cytometry analysis and quantification of the number of GFP‐INS‐1 cells, whose viability remained 41.4% in the TH‐treated group at day 30 but decreased to 4.6% in the PBS group at day 20 (Figure 3C,D). These data proved that the xenografted cells survived longer in the TH‐remodeled testicle. Compared with other studies,^1^, ^2^, ^33^ we found that different approaches could control the blood glucose level for varying lengths of time; this variance may largely depend on the cell types used—for instance, INS‐1 cells seemed less powerful than primary islet cells or stem cell‐derived beta cells in this regard. This shortcoming of insulinoma is reasonable and can probably be overcome by further optimizing the cell source or increasing the times of injection. Nevertheless, the great advantages of these cells, including the convenience of acquisition, fast proliferation, and robust function, offer new and alternative possibilities for solving the key challenges in tissue regeneration.

Histological analysis (H&E staining) further indicated that the transplanted beta cells maintained their normal morphology and developed into islet‐like structures in the TH‐remodeled testicle 20 days after injection; however, this morphology was not observed in the PBS‐treated group, even if the two groups were injected with the same number of cells (Figure 3E). Fluorescence images of the GFP‐INS‐1 cells consistently confirmed the difference between these two groups (Figure 3F). Moreover, IF staining of the testicle showed that the INS‐1 cell clusters formed in the TH group highly expressed NKX 6.1 (a key homeobox transcription factor persistent in pancreatic cells) and MAFA (a critical transcription factor for beta‐cell function) and secreted insulin and C‐peptide (Figure 3G).^34^, ^35^ The expression of these beta‐cell markers at the transcriptional level was also confirmed to be 5–150 times higher in the TH group than in the PBS group (Figure 3H). The outcomes suggest that the TH‐remodeled testicle supports the prolonged survival of xenogeneic beta cells, their formation of a normal, islet‐like morphology, and consequently their production of insulin in the host.

### 2.4 Transplanted Rat Beta Cells Control the Blood Sugar Level in Mice

**Figure 4:**
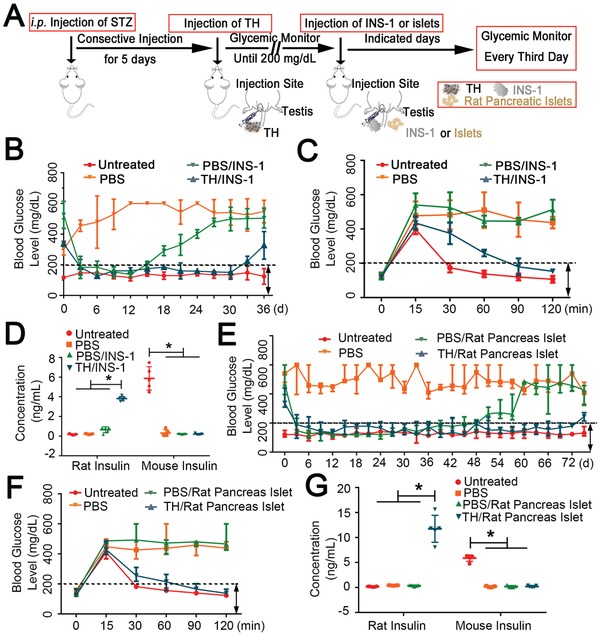
Transplanted rat beta‐cell function to control blood sugar levels in mice. A) Schematic diagram of diabetes reversal by intratesticular injection of INS‐1 or rat pancreas islet cells. B) Blood glucose levels monitored every third day after the diabetic mice were generated, and INS‐1 cells were subsequently injected into TH‐remodeled or PBS‐treated testicles (*n* = 3 per group). C) Blood glucose levels monitored at 0, 15, 30, 60, 90, and 120 min after glucose stimulation (2 g kg^−1^) during the IPGTT 20 days after the injection of INS‐1 cells (*n* = 3 per group). D) Insulin level in the serum of different rat and mouse groups measured at 30 min during the IPGTT as shown in panel (C) (*n* = 3 per group). E) The blood glucose levels were monitored every third day following the generation of diabetic mice and subsequent injection of rat pancreas islet cells into TH‐remodeled or PBS‐treated testicles (*n* = 4 per group). F) Blood glucose levels monitored at 0, 15, 30, 60, 90, and 120 min after glucose stimulation (2 g kg^−1^) during the IPGTT 60 days after the injection of rat pancreas islet cells (*n* = 4 per group). G) Insulin level in the serum of different rat and mouse groups measured at 30 min during the IPGTT treated as shown in panel (F) (*n* = 4 per group). The results are shown as the mean ± SD. *p < 0.05 after ANOVA with Dunnett's tests.

Finally, we tested the function of the xenografted beta cells in the mice, focusing on the ability of these cells to modulate blood glucose level and reverse hyperglycemia. We prepared type I diabetic mice and implanted INS‐1 cells into their testicles using the same protocol as that described above (**Figure** **4**A). The transplanted INS‐1 cells efficiently lowered the blood glucose level in the mice with TH‐remodeled testicles, and this effect was maintained for 30 days. By contrast, in the PBS‐treated mice, these cells could reverse the blood glucose to normal for only 10 days, after which the mice immediately regained hyperglycemia (Figure 4B). Moreover, we tested the response of the grafted INS‐1 cells to a glucose challenge mounted by an intraperitoneal glucose tolerance test (IPGTT) 20 days after injection. The mice were fasted overnight and intraperitoneally injected with glucose before their blood glucose level was monitored at the indicated time points. The outcomes showed that the level of blood glucose was controlled well in the mice with TH‐remodeled testicles, suggesting that INS‐1 cells secreted insulin sensitively in response to glucose (Figure 4C). Importantly, the insulin in the serum of the TH‐remodeled mice 30 min post‐IPGTT was of rat origin—, i.e., it was produced by the xenografted INS‐1 cells rather than by the host mice (Figure 4D). By contrast, INS‐1 cells transplanted to the PBS group failed to respond to the glucose challenge.

INS‐1 cells are immortalized cancerous beta cells. In the mouse body, they survive, develop into islets, and function to secrete adequate insulin, opening new possibilities to resolve the issue of cell transplantation source. Nevertheless, we were interested in whether normal rat pancreatic islets would also function in TH‐remodeled mice. Therefore, we isolated rat pancreatic islets and injected them into the mouse testicle in the same way as that for injecting INS‐1 cells. Similarly, the transplanted rat islets effectively ameliorated hyperglycemia in the TH‐remodeled diabetic mice for 72 days, which was 24 days longer than in the PBS‐treated mice (Figure 4E), and still responded to the glucose challenge 60 days after injection (Figure 4F,G). By contrast, pancreatic islets grafted into the PBS‐treated group failed to change the hyperglycemia condition. The length of time for which glycemic levels in the TH‐remodeled group were normal was 1.6‐fold longer than that in the PBS group (72 vs 45 days). This is a positive result, and further improvement is possible. For instance, increasing the frequency of injection for testicle remodeling can potentially maintain immune tolerance for a longer time, which may consequently give rise to a longer period of blood glucose control.

In summary, these data demonstrate that TH‐remodeled testicles can protect both xenogeneic insulinoma cell lines and primary islet tissue from immune rejection. The testicles functioned longer to produce adequate insulin to control the blood sugar level in the mice. Despite the xenogeneic nature of these functional cells, these trans‐species “guests” demonstrated native morphology and desirable functions for a relatively extended period, which rely upon and is a consequence of successful TH remodeling.

## 3 Conclusion and Discussion

In this study, we showed an innovative and feasible strategy to overcome the two most important challenges in pancreatic islet transplantation: graft rejection and a limited number of therapeutic cells/tissue.^3^ This strategy comprises two steps: i) remodeling of the testicle with a TH and ii) implantation of immortalized xenogeneic cells into the remodeled testicle. The first step creates a stable immune‐suppressive and tolerant environment that is desirable for avoiding graft rejection, while the second step allows for the use of XCCs as the source of therapeutic cells—which may fundamentally solve the longstanding problem of shortage of cell source.^36^ Our exploration opens up the possibility of using xenogeneic insulinoma cell lines as seed cells for pancreatic islet regeneration, which may further inspire the design of new, cell‐based strategies for diabetes treatment.

Immune rejection is the main threat to pancreatic islet transplantation, causing the loss of 50–75% islets shortly after grafting. Conventional approaches focus on the provision of an immunoisolation capsule or systemic administration of immunosuppressive drugs,^5^, ^6^ both of which have substantial side effects. In comparison, our strategy aims to address this issue fundamentally by changing an existing organ of the body—the testicle—into an immunotolerant environment for transplantation. We learned from previous studies (and then validated in this study) that remodeling of the testicle is necessary.^22^ Accordingly, we utilized the TH to change the inner structure and physiological features of the mouse testicle with the aim of enhancing and prolonging its immunosuppressive characteristics. Our strategy proved effective, showing not only the enhancement of immunosuppression but also the establishment of the immunotolerance of the remodeled testicle to xenogeneic cell transplantation. First, TH remodeling effectively increased both the proportion and number of antiinflammatory macrophages (M2), which are the major immune cells mediating immunosuppression.^12^, ^14^, ^25^ Their activity is fundamental to avoid acute inflammation and immune rejection. Second, we observed the reduced secretion of IgM and IgG,^32^, ^37^ the lowered ratio of cytotoxic CD 8^+^ T cells,^27^, ^29^ as well as an increased number of immunosuppressive CD 25^+^/Foxp3^+^ Treg cells.^13^, ^26^ All these data highlighted the establishment of immunotolerance, which is key to accommodating xenogeneic cells and supporting their long‐term function. We emphasize the second point because previous studies illustrated that the native, immunosuppressive niche of the testicular tissue was insufficient to support xenograft function.^38^, ^39^ Direct engraftment into the testicle led to very little success due to the short period of engraftment settlement and the low efficiency with which transplanted islets regulated blood glucose.^20^, ^21^ In summary, our data highlighted the necessity and efficacy of TH remodeling.

Successful minimization of immune rejection has opened up the possibility of using immortalized cells, such as cancer cells, for therapeutic transplantation. Indeed, these cells grow rapidly and function properly,^23^, ^24^, ^40^ which desirably meet the expectation for tissue transplantation. Our data showed that INS‐1 cells seeded in the TH‐remodeled testis smoothly settled down, demonstrated extended survival and, importantly, developed into a Langerhans‐like cell cluster in the intratesticular site.^41^ A normal morphology is important to the function of tissue cells. Indeed, the xenograft cells abundantly expressed beta‐cell markers NKX6.1 and MAFA and effectively secreted insulin. Correspondingly, when we transplanted normal rat pancreatic islets (not insulinoma) into the mouse testicle, they also showed favorable therapeutic potential. Therefore, our study uncovers the underestimated potential of XCCs: these cells are traditionally not considered for therapeutic use, but we show the possibility of using and transplanting them with an ultimate goal of treating type I diabetes.^42^ Further translation of this strategy may help to address the longstanding deficiency of transplantable beta cells.^43^ Engineering of INS‐1 or other insulinoma cells, as well as increasing the time of TH injection to remodel the tissue, may be an effective approach to improve cell performance, which is necessary for the next‐stage translation of this approach before clinical trials.

Our strategy has also taken into account the safety issue throughout the design and implementation of the experiments. First, we rigorously examined in this and previous studies that although TH efficiently modulates the local immune response in the testicle, it has little influence on the global immune system.^22^ Next, despite their cancerous nature, INS‐1 cells displayed a normal islet‐like morphology and the expected insulin‐producing activity, without showing any abnormity. Furthermore, the intentionally produced and sharp contrast between the immunotolerant niche inside the testicle and the immunocompetent environment of the entire mouse body (except the remodeled testicle) is crucial. As one of the most creative designs in this strategy, this “immunological contrast” effectively confined the transplanted cells strictly within the testicle. As such, the rat beta cells demonstrated both effective function (regulating blood glucose levels) and high in vivo safety in the remodeled mouse testicle.

Although we have demonstrated the potential of our approach for pancreatic islet regeneration, we believe that much work lies ahead in terms of application. First, we should evaluate more comprehensively the safety of the strategy in a clinically relevant model. In‐depth analyses are expected for i) changes to the primary functions of the testicles, ii) the TH interference with the immune system outside the testicles, and iii) the confinement of the implanted xenograft cell lines in the testicles. Second, to meet the demand on the quantity of cells for clinical transplantation (≈1.5–3 × 10^9^ beta cells), a substantially larger number of cells should be prepared. This requirement further highlights the importance of using immortalized cell lines such as INS‐1. Finally, further screening or genetic modification can be performed to produce more powerful beta cells that are comparable to mature human beta cells in response to glucose.

## 4 Experimental Section

*Animals*: Male ICR mice (20 ± 2 g) of the same ground were obtained from Model Animal Research Centre of Nanjing University (Nanjing, China). All animals had free access to rodent chow and water, and were treated in strict accordance with the institutional ethical regulation on animal experiments. Animal protocols were reviewed and approved by the Animal Care and Use Committee of Nanjing University, and conformed to the Guidelines for the Care and Use of Laboratory Animals published by the National Institutes of Health.

*Extracts from TH*: The tumor homogenate was obtained according to the method reported in the published literature.^22^ Briefly, to generate the heterotopic tumor model, mouse sarcoma cell line S180 cells (1 × 10^6^) were injected subcutaneously into the left arm pits of the animals. Mice bearing implanted tumors were sacrificed when the sizes of the implanted tumors reached about 0.5 cm. The tumors were removed, immersed in ice‐cold PBS, minced, and washed with the same solution. The mince was homogenized with a teflon/glass homogeniser. The homogenate was centrifuged at 12 000 rpm for 10 min at 4 °C. The pellets were discarded and the supernatant was collected as TH.

*Proteomics Analysis of TH Composition*: The composition of TH was detected by label‐free LC‐MS and ProteinPilot 4.5 software (AB SCIEX) adhered to a method described previously.^44^ Then, LC‐MS profile of TH was analyzed by Reactome Pathway (https://reactome.org/). Cluster analysis of proteins related to the top 15 enriched pathways were performed according to Gene Ontology database (http://www.geneontology.org/). The analysis was performed with standard enrichment computation method.

*Culture and Preparation of INS‐1 Cells*: Rat insulinoma beta cell INS‐1 was obtained from Stem Cell Bank, Chinese Academy of Sciences (Shanghai, China). GFP stably expressing INS‐1 cells (GFP‐INS‐1) were sorted in the presence of puromycin (10 µg mL^−1^; Sigma‐Aldrich, St. Louis, MO, USA) after the cells were transfected with lentivirus expressing GFP and carrying puromycin‐resistant gene. Cells were cultured in RPMI 1640 medium containing 10% fetal bovine serum (FBS) (Thermo Scientific), 50 × 10^−6^ m 2‐mercaptoethanol (β‐ME, Sigma‐Aldrich), 1 × 10^−3^ m glutamine (Thermo Scientific) and 1 × 10^−3^ m Hepes (Thermo Scientific), harvested at ≈80% confluency, washed twice with PBS, counted, and resuspended in PBS for use.

*Isolation of Rat Pancreatic Islets*: After decapitation, abdominal cavity of rat was opened up to expose the bile duct. The common bile duct at the junction with the duodenum was carefully clamped off with a hemostat to prevent flow into the duodenum. Collagenase V (1 mg mL^−1^; Sigma‐Aldrich) in Hanks' balanced salt solution (HBSS) was injected into the bile duct with a 27 gauge needle away from the liver and toward the pancreas until the entire pancreas was expanded (≈8 mL). The pancreas was then dissected away from the rest of the animal and placed into a 15 mL tube with 10 mL of collagenase solution and kept in a water bath (37 °C) for 14 min. The reaction was stopped by adding ice‐cold HBSS supplemented with 10% FBS and then the solution was strained to separate the undigested tissue. To separate islets, the pellets of digested tissue following centrifugation were resuspended in Histopaque (Sigma‐Aldrich) and subsequently HBSS was slowly added.^45^ The gradients were then centrifuged at 2000 rpm for 10 min, after which the islets were washed twice with HBSS. Islets were further purified by observation and handpicking under light microscopy.

*Remodel Testicle with TH*: TH or PBS in same volume was injected into one testicle of male ICR with 31 G needles (BD Biosciences) for one time (500 µL kg^−1^). Further analysis was performed on the testicles 5 days later.

*Implantation of INS‐1 Cells or Islets into the Remolded Testicle*: INS‐1 cells (5 × 10^6^) or rat pancreatic islets (500 islet equivalent quantity, IEQ) in 30 µL PBS were injected into testicle 5 days post the TH or PBS treatment. At the indicated time points, testicles with intratesticular injections were harvested for further analysis.

*Bioluminescence Imaging*: Testicles of the mice implanted with GFP‐INS‐1 cells were monitored by bioluminescence imaging. Briefly, indicated days after injection, the animals were anesthetized with isoflurane and testicles were respectively extracted. GFP bioluminescence was imaged by the IVIS Lumina XR system (PerkinElmer) at an excitation wavelength of 488 nm. The obtained images were analyzed using Velocity 3D Image Analysis Software (PerkinElmer).

*Quantitation of Proteins by Enzyme Linked Immunosorbent Assay (ELISA)*: Serum or supernatant of testicle homogenate was collected and frozen at −80 °C before use. The levels of IgG, IgM, testosterone, rat insulin, mouse insulin, anti‐inflammatory factors (Activin‐A, IL‐10 and TGF‐β1) and proinflammatory factors (IL‐6, IL‐1β, and TNF‐α) were measured using ELISA quantitation kits (Biolegend) according to the manufacturer's instructions.

*Flow Cytometry Analysis*: Testicle tissues were digested to generate a single‐cell suspension, which was then blocked with 1% bovine serum albumin and incubated with the fluorescence‐conjugated monoclonal antibodies specific for the following cell surface markers in the dark for 30 min at 4 °C: CD 11c, CD 49b, CD 45RB, CD 206, CD 86, F4/80, CD 4, CD 8, CD 25, and Foxp3 (eBioscience, MA, USA). The samples were centrifuged at 400–500 × *g* for 5 min at 4 °C to remove unbound antibody. After rinsing for 3 times, each sample was resuspended for analysis using a BD FACS Calibur (BD Biosciences). Unconjugated antibodies and IgG controls were run in parallel to set the background. Besides, to analyze the growth of GFP‐INS‐1 cells as xenografts, the testicles with intratesticular injection were digested and subjected to FACS. The number of GFP‐positive cells was calculated by multiplying the total cell number in testicle and the percentage of the positive cells.

*RNA Isolation and Quantitative Real‐time Polymerase Chain Reaction (qRT‐PCR)*: Total RNA from tissues was extracted by using Trizol (Life Technologies). qRT‐PCR was performed by using a SYBR Prime Script RT‐PCR Kit (Takara Bio) in an ABI 7300 Fast Real‐time PCR System (Applied Biosystems). Each sample was analyzed in triplicates and repeated for three or four independent assays. The level of each gene was normalized to that of β‐actin. Primers used are listed below (Invitrogen, Carlsbad, CA, USA).

**Table:** 

Name	Forward (5′‐3′)	Reverse (5′‐3′)
Insulin	GGAAACCATCAGCAAGCAGGTC	AAGAATCCACGCTCCCCACAC
MAFA	GTCGGATGACCTCCTCCTTGC	CACTCTGCCCACCATCACCAT
NKX 6.1	CCTCCGCTGGATTTGTGCTTT	GGCGGACCAAGTGGAGAAAGA
C‐peptide	CCACCATGAATAGTGAGGAGCAGT	GCACGCAGGGGGATTAGCA
CCR‐7	TTCAACATCACCAATAGCAG	GAAGGCATACAAGAAAGGG
iNos‐2	CAGCTGGGCTGTACAAACCTT	CATTGGAAGTGAAGCGTTTCG
TNF‐α	AGGCGGTGCTTGTTCCTCA	GTTCGAGAAGATGATCTGACTGCC
INF‐γ	TTTTCAGCTCTGCATCGTTTTGGGT	CCTTGAAACAGCATCTGACTCCTT
β‐actin	GGTGTGATGGTGGGAATGGG	ACGGTTGGCCTTAGGGTTCAG

*The Diabetic Mice Model*: The type 1 diabetic mice were prepared by intraperitoneal injection of streptozotocin (STZ, 45 mg kg^−1^ in acetate phosphate buffer, pH 4.5; Sigma‐Aldrich) to ICR male mice for consecutive 5 days. Blood glucose levels were monitored every third day; only mice with a blood glucose level above 200 mg dl^−1^ for consecutive tests were considered diabetic and received subsequent treatments.

*Glycemic Monitoring after Injection of INS‐1 Cells or Rat Islets*: After the diabetic mice were generated, INS‐1 cells or rat pancreatic islets were injected into PBS or TH treated testicles. Next, the levels of blood glucose were monitored every third day following the xenogeneic injection with glucose meters (Roche). Monitoring continued until the end of experiment, when the mice were euthanized and tissues retrieved.

*IPGTT*: To detect the xenograft's responses to glucose challenge, IPGTT was conducted 20 days or 60 days after the injection of INS‐1 cells or rat pancreatic islets into testicles treated with PBS or TH. This assay could further assess the xenograft's reversal capacity in response to a glucose bolus. Briefly, animals were fasted overnight before receiving an intraperitoneal glucose bolus (2 g kg^−1^, Sigma‐Aldrich). The levels of blood glucose were monitored at 0, 15, 30, 60, 90, and 120 min after injection. Blood samples were also collected to measure the glucose‐stimulated secretion of rat and mouse insulin at 30 min. In these experiments, the blood was centrifuged for 20 min at 4000 × g and the serum was stored at −80 °C until use.

*Histological Analysis*: The tissue samples were collected, frozen at optimal cutting temperature medium (Leica), and cut into sections for H&E staining according to the manufacturer's instructions (KeyGEN BioTECH). The stained sections were photographed at different magnifications. Under a standard light microscopy, the tissues were randomly examined. Meanwhile, the frozen tissue sections for immunofluorescent staining were fixed with 4% paraformaldehyde (Sigma‐Aldrich) and stained with primary antibody at 4 °C overnight. The primary antibodies included anti‐mouse F4/80, CD 8, NKX 6.1, MAFA, C‐peptide, and insulin (Abcam). Next, the sections were incubated with secondary antibody Alexa Fluor (Life Technologies) for 1 h at room temperature, followed by 4,6‐diamidino‐2‐phenylindole (DAPI) for nuclear staining. All fluorescence including GFP bioluminescence were captured with a C2+ confocal microscope (Nikon) and analyzed using Nis‐element advanced research software (Nikon).

*Statistical Analysis*: The results are expressed as mean ± standard deviation (SD). Data were statistically analyzed using Prism software (GraphPad) and assessed for normality or homogeneity of variance with Shapiro‐Wilk‐test and Barlett test. Differences between multiple groups were compared using one‐way or two‐way analysis of variance (ANOVA) with Dunnett's tests. Differences between two groups were evaluated using the unpaired Student's *t‐*test. A value of *P* < 0.05 was considered significant.

## Conflict of Interest

The authors declare no conflict of interest.

## Supporting information

Supplementary

Supplementary
