# Supplementary material for: Growing Trans‐Species Islets in Tumor Extract‐Remodeled Testicles
Source: Adv Sci (Weinh). 2019 Jan 13;6(6):1801694. doi: 10.1002/advs.201801694 (PMC6425427; doi:10.1002/advs.201801694)
Supplement: Supplementary file 1 — Supplementary [file ADVS-6-1801694-s001.pdf]

Copyright WILEY-VCH Verlag GmbH & Co. KGaA, 69469 Weinheim, Germany, 2019.

ADVANCED SCIENCE
Open Access

Supporting Information

for *Adv. Sci.,* DOI: 10.1002/advs.201801694

Growing Trans-Species Islets in Tumor Extract-Remodeled Testicles

Zhenzhen Wang, Xiaying Rui, Junni Qiu, Yiqing Yan, Jingjing Gan, Shang Liu, Lintao Wang, Junfeng Zhang, Chunming Wang,* and Lei Dong**

DOI: advs.201801694R1
Article type: Full Paper

Supplementary Information

Growing Transspecies Islets in Tumor Extract-Remodeled Testicles

Zhenzhen Wang[1,2,3], Xiaying Rui[2], Junni Qiu[2], Yiqing Yan[2], Jingjing Gan[2], Shang Liu[2], Lintao Wang[2,3], Junfeng Zhang[1,2,*], Chunming Wang[3,*], Lei Dong[2,*]

1. Affiliated Drum Tower Hospital, Medical School of Nanjing University, Nanjing, Jiangsu 21093, China.
2. State Key Laboratory of Pharmaceutical Biotechnology, School of Life Sciences, Nanjing University, Nanjing, Jiangsu 210023, China.
3. State Key Laboratory of Quality Research in Chinese Medicine, Institute of Chinese Medical Sciences, University of Macau, Taipa, Macau SAR.

* Corresponding Authors: L.D., Email: leidong@nju.edu.cn ; C.M.W., Email: cmwang@umac.mo ; J.F.Z., Email: jfzhang@nju.edu.cn .

Table S1. Analysis of the protein components in TH by LC-MS.
Table S2. Analysis of pathways significantly enriched by the TH proteins.
Figure S1. Proteomics analysis of TH composition.
Figure S2. The levels of testosterone in testicle 5 days after a single injection of TH or PBS.
Figure S3. Representative FACS analysis of main immune cells in testicle 5 days after injection of PBS or TH;
Figure S4. Representative FACS analysis of CD 86^{+}/F4/80^{+} and CD 206^{+}/F4/80^{+} in testicle 5 days after injection of PBS or TH;
Figure S5. Representative FACS analysis of CD 86^{+}/F4/80^{+} in testicles 5 days after injection of INS-1;
Figure S6. Images of insulin immunofluorescent staining for INS-1 in other tissues 5 days after injection of INS-1;
Figure S7. Average fluorescence intensity of bioluminescence imaging of PBS or TH treated testicles injected with GFP-INS-1 at indicated days.

Table S2. Analysis of pathways significantly enriched by the TH proteins. (By Reactome pathway).

Category	Related Pathways (Rank[a])
Immune Response	Immune System (2)<br>Cytokine Signaling in Immune system (5)<br>Innate Immune System (6)<br>Signaling by Interleukins (9)<br>Adaptive Immune System (13)<br>Interleukin-4 and Interleukin-13 signaling (15)
Signaling Response	Signal Transduction (1)<br>Signaling by Receptor Tyrosine Kinases (3)<br>PIP3 activates AKT signaling (8)
Cellular Response or Activation	Platelet activation, signaling and aggregation (11)<br>Cellular responses to external stimuli (12)
Developmental Process	Developmental Biology (4)<br>Extracellular matrix organization (14)
Metabolism Process	Metabolism of proteins (7)<br>Metabolism (10)

a. Rank of each pathway sorted by the enrichment.

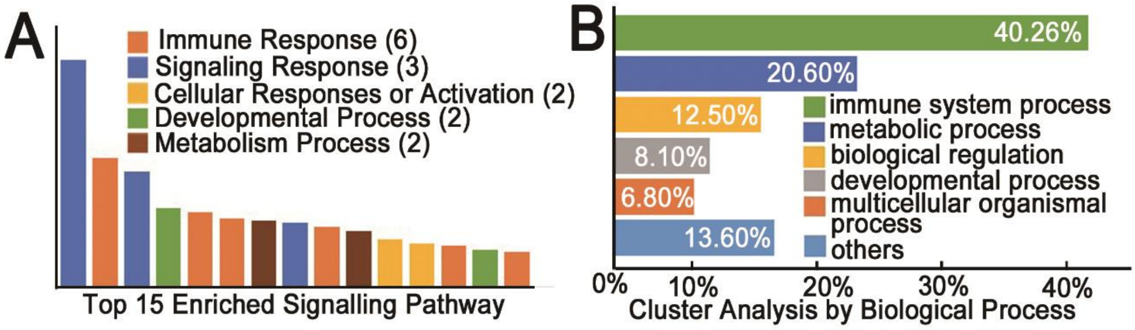

Figure S1. Proteomics analysis of TH composition. (A) Analysis of top 15 pathways significantly enriched by the TH proteins according to Reactome Pathway. (B) Hierarchical cluster analysis of proteins related to top 15 pathways shown in panel (A) according to biological process based on Gene Ontology Database.

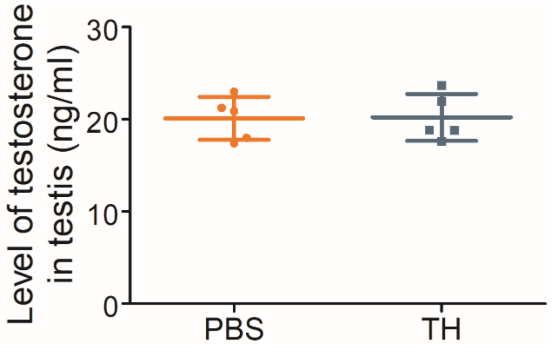

Figure S2. The levels of testosterone in testicle 5 days after a single injection with TH (500 µl/kg) or PBS. Results are shown as mean ± SD (n = 5 mice per group). * $p<0.05$ after ANOVA with Dunnett's tests.

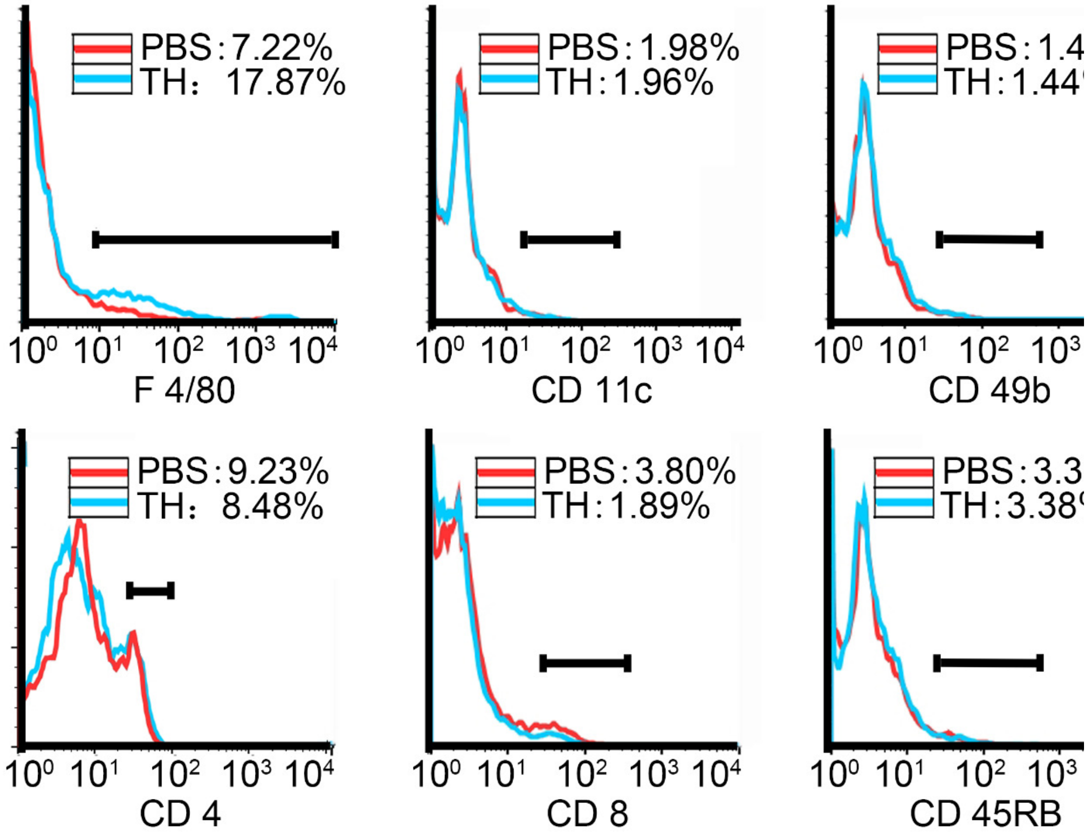

Figure S3. Representative FACS analysis of main immune cells (F4/80-macrophage; CD 11c-DC cells; CD 49b-NK cells; CD 4 T cells, CD 8 T cells; CD 45RB-B cells) in testicle 5 days after single injection of PBS or TH;

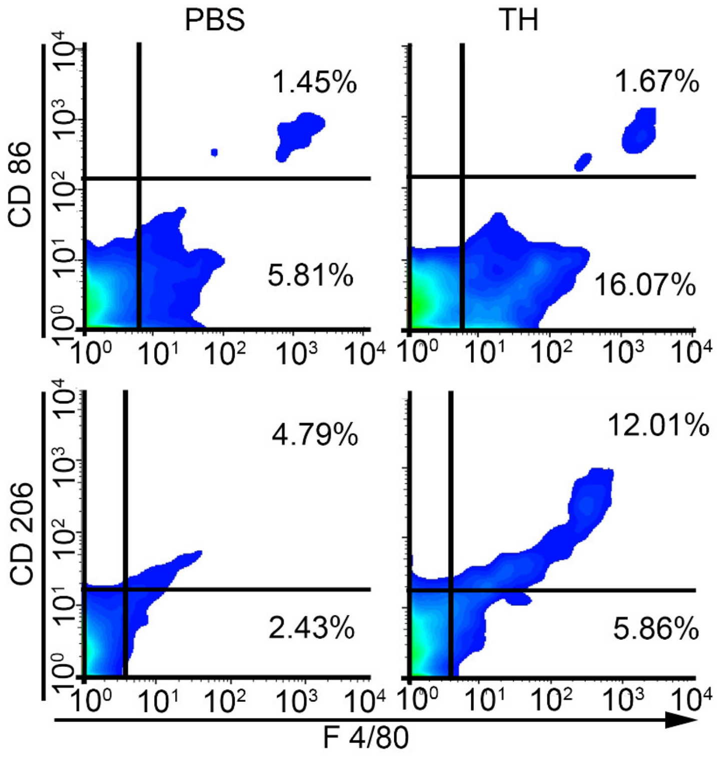

Figure S4. Representative FACS analysis of CD 86$^+$/F4/80$^+$ and CD 206$^+$/F4/80$^+$ in testicle 5 days after single injection of PBS or TH.

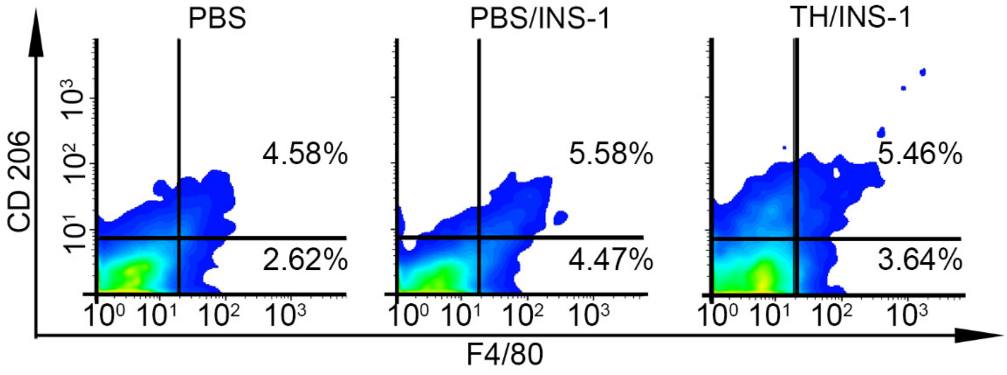

Figure S5. Representative FACS analysis of CD 206$^+$/F4/80$^+$ in PBS or TH treated testicles 5 days after injection of INS-1.

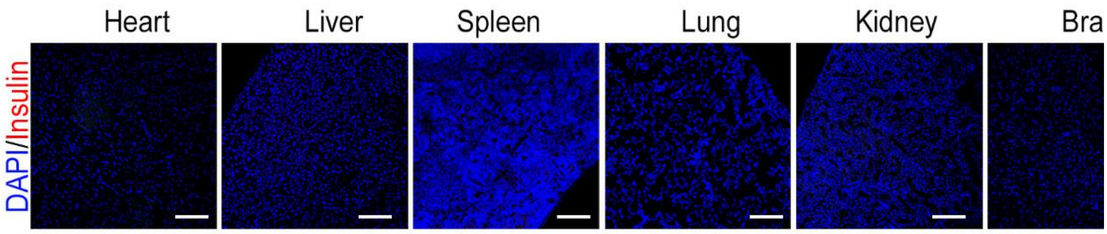

Figure S6. Images of insulin immunofluorescent staining for INS-1 cells in other tissues 5 days after injection of INS-1. Scale bar=100 μm. Images are representative for three independent experiments.

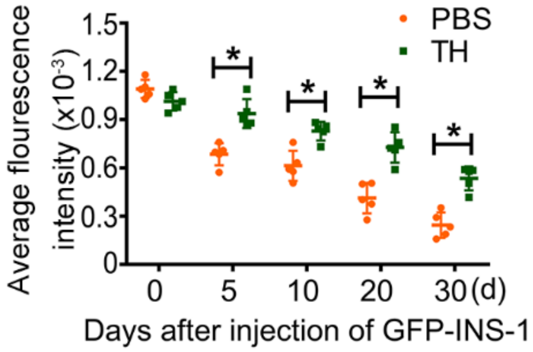

Figure S7. Average fluorescence intensity of bioluminescence imaging of PBS or TH treated testicles injected with GFP-INS-1 at indicated days. Results are shown as mean ± SD (n = 5 mice per group). * $p<0.05$ after ANOVA with Dunnett's tests.
